# Maintenance of prehospital anaesthesia in trauma patients: inconsistencies and variability in practice

**DOI:** 10.1016/j.bjao.2024.100366

**Published:** 2025-01-09

**Authors:** Brad Sheridan, Zane Perkins

**Affiliations:** 1 Hunter Retrieval Service and Department of Anaesthesia, John Hunter Hospital, Newcastle, NSW, Australia; 2 Centre for Trauma Sciences, Blizard Institute, Queen Mary University of London, London, UK; 3 London's Air Ambulance, London, UK

**Keywords:** anaesthesia, Australia, emergency medical services, intubation, New Zealand, United Kingdom

## Abstract

**Background:**

Literature on prehospital anaesthesia predominantly focuses on preparation and induction, while there is limited guidance on anaesthesia maintenance. The hypothesis of this study was that for prehospital trauma patients, protocols and practice for anaesthesia maintenance may vary considerably between services. Hence, we sought to describe the practice of prehospital anaesthesia maintenance for trauma patients in Australia, New Zealand, and the UK.

**Methods:**

An online practice survey of prehospital and retrieval services in Australia, New Zealand, and the UK was conducted from May to September 2022. Branching logic of between five and 140 questions covered services' background information, protocols relating to anaesthesia maintenance, and perceived effectiveness and governance.

**Results:**

Forty-two services were approached with an 81% response rate. While most services (88%) had some form of maintenance protocol, only 14% had one specific for trauma patients. Most services (61%) used a combination of intermittent boluses and continuous infusions. Ketamine and midazolam were the favoured hypnotics, and fentanyl the favoured opioid. However, there was considerable variation in drug selection and dosing, and in the detail contained within protocols. There was high self-reported confidence in effectiveness and governance of anaesthesia maintenance practices.

**Conclusions:**

Protocols for anaesthesia maintenance in prehospital trauma patients show considerable variation in content and detail across the surveyed services. Further consideration of pharmacokinetics and the specific aims of anaesthesia maintenance is warranted. More research is needed to establish the optimal choice of drugs, dosing, delivery, and adjustment criteria for anaesthesia maintenance in prehospital trauma patients.

Trauma-related incidents continue to constitute a significant portion of prehospital services' workload in the Western world.^1^^,^^2^ The evolution of advanced care prehospital teams in these countries has seen a rise in complex interventions in the prehospital setting, including the provision of prehospital emergency anaesthesia (PHEA). Severely injured trauma patients often meet accepted indications for PHEA,^3^, ^4^, ^5^, ^6^, ^7^ however the benefits remain contentious.^7^, ^8^, ^9^, ^10^ Nonetheless, recognition of its dangers and the paramount need for safety when providing PHEA are undisputed.^3^^,^^9^^,^^11^

To this end, PHEA is usually practised within a framework of strict governance and detailed guidelines or standard operating procedures (SOPs).^3^^,^^11^, ^12^, ^13^, ^14^ There is extensive literature on PHEA,^11^^,^^14^ and it is a recognised area of need for ongoing research.^15^^,^^16^ However, while much of the literature focuses on preparation, induction, and airway management for prehospital anaesthesia,^13^^,^^15^^,^^17^, ^18^, ^19^, ^20^, ^21^, ^22^ there are few articles dedicated to the maintenance of anaesthesia post-rapid sequence induction (RSI) in the prehospital setting.^23^, ^24^, ^25^, ^26^, ^27^ Moreover, although certain areas of post-RSI care, such as mechanical ventilation and haemodynamic management, have been identified as areas that require further research, maintenance of anaesthesia post-RSI is often overlooked.^16^, ^17^, ^18^^,^^28^ Consequently, there is limited literature to help guide prehospital clinicians and services on protocols for the optimal maintenance of anaesthesia for prehospital trauma patients.

The underlying hypothesis of this study is that for maintenance of anaesthesia post-RSI in prehospital trauma patients, SOPs lack clear guidance and practice is variable. Hence, the primary aim of this survey is to characterise the state of clinical practice in Australia (AU), New Zealand (NZ), and the UK regarding maintenance of anaesthesia post-RSI during transportation of trauma patients to hospital. Specifically, this practice survey seeks to elucidate whether prehospital and retrieval medicine services have a guideline or SOP on maintenance of anaesthesia post-RSI in prehospital trauma patients, and if so what drug(s) and mode(s) of administration are recommended, and what factors may affect the dosing and timing of these regimens. The practice survey will also seek to elucidate services' governance and perceived effectiveness of maintenance of anaesthesia protocols in prehospital trauma patients.

## Methods

### Study population and design

This was a cross-sectional multinational practice survey with purposive sampling targeting prehospital and retrieval medicine services in AU, NZ, and the UK that perform PHEA for trauma patients. The CROSS (consensus-based checklist for reporting of survey studies) checklist has been followed in the reporting of this survey.^29^

The electronic data capture tool REDCap,^30^^,^^31^ hosted at the Hunter Medical Research Institute, was used to structure survey questions, manage invitations, collect and organise data, and collate and analyse results. The survey used branching logic and comprised a potential total of 140 questions, divided into three sections, with the second section divided into two parts (Supplementary Appendix S1). The first section covered questions on service characteristics and the contextual aspects of any SOPs for maintenance of anaesthesia regimens; the second section sought details on recommended intermittent bolus, continuous infusion regimens, or both; and the third section covered perceived effectiveness and governance. The first and last sections were common to all recipients, while the contents of the second section were individualised based on participants' responses. The expected time to complete the survey was between 5 and 15 min.

### Study conduct, data collection, and analysis

A pre-test of the survey to assess functionality and identify errors was sent by automated email via REDCap to three prehospital and retrieval medicine clinicians. Two of these clinicians work for different UK services and one for an Australian service. While their services were included in the survey, these clinicians were not the responding clinicians for their service in the survey proper. Two rounds of pre-testing were required to guarantee access by international participants and finalise the survey contents.

The survey was conducted online between May and September 2022. Invitations with a unique URL link and code were sent via REDCap to the clinical directors of prehospital and retrieval medicine services in AU, NZ, and the UK. UK contacts were compiled from a registry held by the National HEMS (Helicopter Emergency Medical Service) Research and Audit Forum. As there is no central research body for prehospital and retrieval medicine services in AU and NZ, the AU and NZ contacts were compiled from a combination of personal contacts, introductions, and online searches conducted by the authors. One response was sought from each service, and clinical directors could delegate responsibility for completing the survey by sharing the unique URL with another appropriate clinician within their service.

Automated emails via REDCap were first sent on 18 May 2022, and then two automated follow-up emails were sent at 1-week intervals. One individual follow-up email was sent manually as a final reminder to non-responders and those with incomplete entries. If follow-up emails for incomplete entries led to multiple entries from a single service, only the completed entry was included in the final analysis. The survey closed on 30 September 2022. Subsequently, explanation was sought using individual follow-up email for responses that required clarification during the results analysis and synthesis stage in January and February 2023. The results are presented using simple descriptive statistics and figures. We did not plan to use inferential statistical analyses.

### Ethics and data security

The survey was granted an exemption from research ethics committee review as a ‘negligible risk research activity’ by the Hunter New England Human Research Ethics Committee. Access to the survey results was only possible through REDCap's two-factor authentication process. Survey results were confidential and individual services are not identified.

## Results

### Survey responses

Surveys were distributed to 42 services: 21 in the UK, 17 in AU, and four in NZ. Response rates were 95% (20/21) for the UK, 65% (11/17) for AU, and 75% (3/4) for NZ, resulting in an overall response rate of 81% (34/42). The median time taken to complete the survey was 6 min, with a median of 23 questions answered. Clarifications on responses were sought from five services.

### Maintenance of anaesthesia protocols

Of the 34 responding services, 28 (82%) reported having protocols for prehospital anaesthesia maintenance (Table 1). Most protocols offered general guidance, while only four services (14%) provided a specific protocol for prehospital trauma patients. Among the services with anaesthesia maintenance protocols, 17 (61%) used a combination of intermittent boluses and continuous infusions, six (21%) relied solely on continuous infusions, and five (18%) used only intermittent boluses. No service reported using any other drugs for maintenance other than ketamine, midazolam, and propofol as hypnotics; fentanyl and morphine as opioids; and rocuronium for neuromuscular blockade.

**Table 1:** Summary of maintenance of anaesthesia post-RSI protocols. All, MAP-RSI protocol for all prehospital trauma and medical patients (with or without interhospital patients); AU, Australia; B, bolus; I, infusion; MAP-RSI, maintenance of anaesthesia post-RSI; N, no MAP-RSI protocol; NZ, New Zealand; RSI, rapid sequence induction (of anaesthesia); Trauma, MAP-RSI protocol specific to prehospital trauma patients; Y (C), yes, MAP-RSI protocol contained within prehospital anaesthesia protocol; Y (S), yes, MAP-RSI protocol separate to prehospital anaesthesia protocol.

Service (country)	MAP-RSI protocol	MAP-RSI regimen	Patient population	Maintenance drugs						Notes
				Ketamine	Midazolam	Propofol	Fentanyl	Morphine	Rocuronium	
1 (UK)	Y (C)	I	All	I						
2 (UK)	Y (C)	B	All	B	B		B	B	B	
3 (UK)	Y (C)	B and I	All		B	I	B	B	B	
4 (UK)	Y (C)	B and I	All	I	I		I	I		Boluses also recommended, but no drugs specified
5 (NZ)	Y (C)	B and I	All	I		I	B		B	
6 (UK)	Y (C)	B and I	All			I				Boluses also recommended, but no drugs specified
7 (AU)	Y (C)	B and I	Trauma	B and I	B and I		I			
8 (UK)	N									
9 (AU)	Y (C)	I	All	I	I					
10 (NZ)	Y (C)	I	All	I			I			
11 (UK)	(incomplete duplicate entry deleted)									
12 (UK)	Y (C)	B and I	All							Boluses and infusions recommended, but no drugs specified
13 (NZ)	Y (S)	B and I	All	B			B			Infusions also recommended, but no drugs specified
14 (UK)	N									
15 (UK)	Y (C)	B and I	All	B	B	B and I	B	B		
16 (AU)	N									
17 (AU)	Y (C)	B and I	All					B		Infusions also recommended, but no drugs specified
18 (UK)	Y (S)	B and I	All	B	B		B		B	Infusions also recommended, but no drugs specified
19 (UK)	Y (C)	B and I	All	B	B	I	B		B	
20 (UK)	Y (C)	B	All		B			B		
21 (AU)	Y (C)	B and I	Trauma	B	B		B		B	Infusions also recommended, but no drugs specified
22 (AU)	Y (C)	I	All	I	I	I	I	I	I	
23 (UK)	Y (C)	B and I	All							Boluses and infusions recommended, but no drugs specified
24 (UK)	Y (C)	B	All							No drugs specified
25 (AU)	N									
26 (UK)	Y (C)	B	All	B	B		B			
27 (UK)	Y (C)	B and I	All	B and I		I				
28 (UK)	Y (C)	I	All	I		I			I	
29 (AU)	N									
30 (AU)	Y (S)	B and I	Trauma	B	B and I		B and I			
31 (UK)	Y (C)	B and I	All	I		I	I			Boluses also recommended, but no drugs specified
32 (UK)	Y (S)	I	All	I	I	I				
33 (AU)	Y (C)	I	All		I		I	I		
34 (AU)	N									
35 (UK)	Y (C)	B	Trauma	B	B		B		B	

### Combination of intermittent boluses and continuous infusion regimens

Seventeen services reported using a combination of intermittent boluses and continuous infusions, and their specific dosing recommendations are detailed in Table 2. All 12 services using ketamine for anaesthesia maintenance in combination bolus and infusion regimens also used it for induction. Of the nine services that used midazolam, three also used it for anaesthesia induction. Similarly, of the seven services that utilised propofol, three also used it for induction. Twelve services specified fentanyl in their combination bolus and infusion regimens, and all except one (*n*=11) used fentanyl during induction. While two services recommended morphine for maintenance, only one used morphine during induction. All six services that maintained neuromuscular blockade with rocuronium boluses also used rocuronium during induction.

**Table 2:** Combination of intermittent boluses and continuous infusions dosing regimens. AU, Australia; NZ, New Zealand.

Service (country)	Ketamine regimen(s)	Midazolam regimen(s)	Propofol regimen(s)	Fentanyl regimen(s)	Morphine regimen(s)	Rocuronium regimen(s)	Notes
3 (UK)		Bolus: recommended, but nil guidance on dosing or interval		Bolus: recommended, but nil guidance on dosing or interval	Bolus: recommended, but nil guidance on dosing or interval	Bolus: 50 mg q 30 min	
			Infusion: recommended, but nil guidance on dosing or rate				
4 (UK)	-	-	-	-	-	-	Infusions preferred, however intermittent boluses also recommended for shorter jobs and ‘top-ups’. Nil advice on specific drug, dosing, or interval of boluses
	Infusion: recommended, but nil guidance on dosing or rate	Infusion: recommended, but nil guidance on dosing or rate		Infusion: recommended, but nil guidance on dosing or rate	Infusion: recommended, but nil guidance on dosing or rate		
5 (NZ)				Bolus: 25–50 μg q 5 min		Bolus: 50 mg (no interval)	Weight-based dosing in paediatrics (not specified)
	Infusion: 100–200 mg h^−1^		Infusion: 50–200 mg h^−1^				
6 (UK)	-	-	-	-	-	-	Intermittent boluses recommended, but nil advice on specific drug, dosing, or interval. Propofol infusion rate reduced or drug changed in hypotension
			Infusion: 0–300 mg h^−1^				
7 (AU)	Bolus: recommended, but nil guidance on dosing or interval	Bolus: recommended, but nil guidance on dosing or interval					
	Infusion: 50–100 mg h^−1^	Infusion: 5–20 mg h^−1^		Infusion: 50–200 μg h^−1^			
10 (NZ)	Bolus: 20 mg (no interval) (adult)			Bolus: 20 μg (no interval) (adult)			Mix 200 mg ketamine and 200 μg fentanyl to 20 ml in a single syringe for infusion. ‘Top-up’ boluses PRN of 2 ml (for adults) from same syringe. Alternatively, 30 mg midazolam and 300 μg fentanyl to 30 ml in a single syringe, no ‘top-ups’.
	Infusion: 0–200 mg h^−1^, start at 80 mg h^−1^ (adults); 2 mg kg^−1^ h^−1^ (paeds)	Infusion: 0–20 mg h^−1^, start at 8 mg h^−1^ (adults); 0.2 mg kg^−1^ h^−1^ (paeds)		Infusion: 0–200 μg h^−1^, start at 80 μg h^−1^ (adults); 2μg kg^−1^ h^−1^ (paeds)			
12 (UK)	-	-	-	-	-	-	Both intermittent boluses and infusions (but with an emphasis on infusions) recommended. However nil advice on specific drug, dosing, interval or rate.
	-	-	-	-	-	-	
13 (NZ)	Bolus: 20 mg (no interval)			Bolus: 20 μg (no interval)			Boluses as ‘top-ups’ to infusion(s). Infusion regimen(s) recommended, but nil advice on specific drug(s), dosing or rate
	-	-	-	-	-	-	
15 (UK)	Bolus: recommended, but nil guidance on dosing or interval	Bolus: recommended, but nil guidance on dosing or interval	Bolus: recommended, but nil guidance on dosing or interval	Bolus: recommended, but nil guidance on dosing or interval	Bolus: recommended, but nil guidance on dosing or interval		
			Infusion: recommended, but nil guidance on dosing or rate				
17 (AU)						Bolus: 1–2 mg kg^−1^ (no interval)	Infusion regimen(s) recommended, but nil advice on specific drug(s), dosing or rate
	-	-	-	-	-	-	
18 (UK)	Bolus: 0.1 mg kg^−1^ q 10min	Bolus: 0.05 mg kg^−1^ (no interval)		Bolus: 0.5 μg kg^−1^ (no interval)		Bolus: 0.5 mg kg^−1^ (no interval)	Infusion regimen(s) recommended, but nil advice on specific drug(s), dosing or rate
	-	-	-	-	-	-	
19 (UK)	Bolus: 10–15 mg (nil interval)	Bolus: 1–2 mg (no interval)		Bolus: 25 μg (no interval)		Bolus: 0.5 mg kg^−1^ q 20–30 min	
			Infusion: 1.2–4.0 mg kg^−1^ h^−1^				
21 (AU)	Bolus: recommended, but nil guidance on dosing or interval	Bolus: recommended, but nil guidance on dosing or interval		Bolus: recommended, but nil guidance on dosing or interval		Bolus: recommended, but nil guidance on dosing or interval	Infusion regimen(s) recommended, but nil advice on specific drug(s), dosing or rate
	-	-	-	-	-	-	
23 (UK)	-	-	-	-	-	-	Both intermittent boluses and infusions recommended. However nil advice on specific drug, dosing, interval or rate.
	-	-	-	-	-	-	
27 (UK)	Bolus: recommended, but nil guidance on dosing or interval						
	Infusion: 1 mg kg^−1^ h^−1^		Infusion: 1–4 mg kg^−1^ h^−1^				
30 (AU)	Bolus: recommended, but nil guidance on dosing or interval	Bolus: 2–3 mg (no interval)		Bolus: 10–40 μg (no interval)			Mix midazolam and fentanyl in a single syringe for infusions
		Infusion: >5 mg h^−1^		Infusion: >50 μg h^−1^			
31 (UK)	-	-	-	-	-	-	Intermittent boluses recommended, but nil advice on specific drug, dosing, or interval
	Infusion: 1–4 mg kg^−1^ h^−1^		Infusion: 1–6 mg kg^−1^ hour^−1^	Infusion: 0–6 μg kg^−1^ h^−1^			

None of the services provided specific recommendations for the timing of the first maintenance bolus dose of ketamine, midazolam, propofol, fentanyl, or morphine. Of the six services recommending rocuronium boluses, only two (33%) specified the timing of the first maintenance dose, at 20–30 min and 30 min post-RSI, respectively. No service specified the timing of the last bolus dose before handover. Regarding infusions, three services (18%) began infusions immediately after induction, one (6%) started between induction and loading the patient into the helicopter, and six (35%) left the decision to start infusions at the clinician's discretion. The remaining services (59%) provided no guidance on timing of commencement of maintenance infusion(s).

Ten of the 17 services recommended adjusting doses based on clinical criteria, such as haemodynamic stability, indicators of sedation level (e.g. lacrimation, pupil size), patient age, and existing comorbidities.

### Continuous infusion-only regimens

Six services reported using continuous infusion protocols, each specifying a choice of drug(s) and recommended infusion rate. The specific dosing recommendations are summarised in Table 3. All services that used ketamine (*n*=5) and fentanyl (*n*=3) for maintenance by infusion also used these drugs for induction. Only one of the three services recommending propofol infusions also used propofol for induction, while only one of the two services recommending rocuronium infusions also used rocuronium during induction. Four services recommended midazolam and two services recommended morphine for maintenance by continuous infusion but none of these services used these drugs for induction.

**Table 3:** Continuous infusions-only dosing regimens. AU, Australia.

Service (country)	Ketamine regimen	Midazolam regimen	Propofol regimen	Fentanyl regimen	Morphine regimen	Rocuronium regimen	Notes
1 (UK)	1 mg kg^−1^ h^−1^						
9 (AU)	1–2 mg kg^−1^ h^−1^	5–10 mg h^−1^		50–100 μg h^−1^			
22 (AU)	0.25–1 mg kg^−1^ h^−1^	0.02–0.08 mg kg^−1^ h^−1^	1–12 mg kg^−1^ h^−1^	0.3–1.2 μg kg^−1^ h^−1^	0.02–0.08 mg kg^−1^ h^−1^	0.3–0.4 mg kg^−1^ h^−1^	Option to mix morphine and midazolam in single syringe
28 (UK)	0.6–2.4 mg kg^−1^ h^−1^		1–5 mg kg^−1^ h^−1^			0.3–0.9 mg kg^−1^ h^−1^	Ketamine in lieu of propofol in cardiovascular instability
32 (UK)	2.5 mg kg^−1^ h^−1^	0.0625 mg kg^−1^ h^−1^	0–500 mg h^−1^				Mix 200 mg ketamine and 5 mg midazolam to 20 ml in a single syringe. Infuse at (weight in kg/4) mlh^−1^
33 (AU)		>5 mg h^−1^		>50 μg h^−1^	>5 mg h^−1^		Mix 50 mg midazolam with either 500 μg fentanyl or 50 mg morphine to 50 ml in a single syringe. Start at 5 ml h^−1^

Regarding the timing of infusions, three services (50%) started at least one hypnotic infusion immediately post-induction, one service (17%) recommended starting between induction and loading the patient into the helicopter, and two services (33%) left the timing to clinician discretion. Both services using rocuronium infusions also left the timing of the start of these infusions to clinicians' discretion. Four of the six services recommended dose adjustments based on clinical criteria, including haemodynamic stability and the initial level of consciousness.

### Intermittent bolus-only regimens

Five services reported using an intermittent bolus regimen, and their specific dosing recommendations are summarised in Table 4. None of the services that recommended the use of midazolam (*n*=4) or morphine (*n*=2) for ongoing maintenance by intermittent bolus used these drugs for induction of anaesthesia, while all services that recommended ketamine (*n*=3), fentanyl (*n*=3), and rocuronium (*n*=2) for bolus-only maintenance also used these drugs for induction.

**Table 4:** Intermittent bolus-only dosing regimens.

Service (country)	Ketamine regimen	Midazolam regimen	Propofol regimen	Fentanyl regimen	Morphine regimen	Rocuronium regimen	Notes
2 (UK)	Recommended, but nil guidance on dosing or interval	<0.1 mg kg^−1^ (no interval)		Recommended, but nil guidance on dosing or interval	<0.1 mg kg^−1^ (no interval)	0.5 mg kg^−1^ (no interval)	
20 (UK)		1 mg q 10–20 min			1 mg q 10–20 min		
24 (UK)							Nil advice on specific drug, dosing or interval
26 (UK)	Recommended, but nil guidance on dosing or interval	Recommended, but nil guidance on dosing or interval		Recommended, but nil guidance on dosing or interval			
35 (UK)	Recommended, but nil guidance on dosing or interval	Recommended, but nil guidance on dosing or interval		Recommended, but nil guidance on dosing or interval		Recommended, but nil guidance on dosing or interval	

None of the services gave guidance on the timing of the first dose post-induction or the last dose before handover. Four of the five services recommended dose adjustments based on clinical criteria, including haemodynamic stability, lacrimation, and more general ‘clinical parameters’ or ‘clinical judgement’.

### Perceived effectiveness and governance

**Fig 1:**
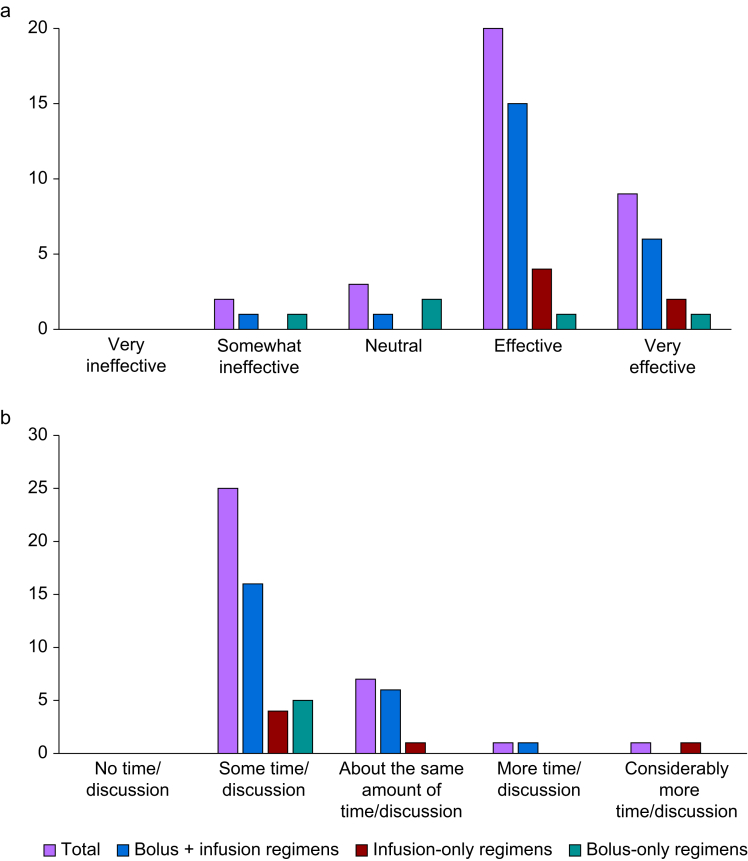
(a) Self-reported effectiveness of recommended maintenance regimens. (b) Time spent in governance meetings discussing post-RSI anaesthesia/sedation. Bolus + infusion regimens,  Infusion-only regimens,  Bolus-infusion regimens,  Total.

Of the 34 services that responded to the survey, the majority considered their regimen to be either effective (*n*=20) or very effective (*n*=9) in providing uninterrupted, adequate levels of anaesthesia/sedation for prehospital trauma patients post-RSI, and there was no discernible pattern between the different types of regimen (Fig. 1a). With respect to governance, the majority of services reported that, relative to the RSI itself, they dedicated some (*n*=25) or about the same (*n*=7) amount of time/discussion in governance meetings to post-RSI sedation practices, with no discernible pattern between the different types of regimen (Fig. 1b).

## Discussion

This multinational practice survey demonstrated that in services providing PHEA to trauma patients, there was considerable variability in both the content and detail of protocols governing maintenance of anaesthesia post-RSI. Despite this, most services were confident in the effectiveness of their maintenance of anaesthesia protocols and spent at least some time discussing their maintenance practices in governance meetings.

Compared with other surveys outlining drugs used for PHEA in UK prehospital services,^13^^,^^22^ the results of our survey help to describe current practice specific to maintenance of anaesthesia post-RSI in more detail regarding drug choice, dosing, timing, and method of delivery. Further, we sought to concentrate on prehospital trauma patients, as the pathophysiology of prehospital medical patients differs such that comparison may be unreasonable.^22^ A 2009 UK survey by Cowan and colleagues^13^ identified that propofol, ketamine, or midazolam, with or without an opioid, were used for maintenance of anaesthesia in patients receiving PHEA. At that time in the UK, the opioids carried were morphine, fentanyl, or alfentanil. Further, in 2009 UK services carried vecuronium, rocuronium, pancuronium, or atracurium as their non-depolarising neuromuscular blocking drugs. However, the 2009 survey by Cowan and colleagues^13^ gave no indication of drug dosing, timing, or means of delivery. More recently, Hodkinson and Poole^22^ published a 2023 survey (contemporaneous to ours) examining PHEA induction regimens by UK HEMS services. In results broadly consistent with ours, they found that services preferred bolus regimens (including ketamine, midazolam, fentanyl, morphine) for maintenance (37%), but that propofol (32%), and to a lesser extent ketamine (16%), infusions were also used. Similarly, clinician discretion was a common theme in both our results and those of Hodkinson and Poole.^22^ However, as with Cowan and colleagues,^13^ the results of Hodkinson and Poole^22^ gave no indication of dosing or timing of maintenance regimens.

Much like for induction of anaesthesia,^22^ our results showed that across UK, AU, and NZ, midazolam and ketamine were the principal hypnotics used for maintenance, that morphine and fentanyl were the only opioids, and rocuronium the only neuromuscular blocking drug. While propofol infusions are still used, their use may be declining in the UK.^13^^,^^22^ Alfentanil is no longer used during induction or maintenance of anaesthesia,^13^^,^^22^ and services no longer use vecuronium, pancuronium, or atracurium for maintenance of neuromuscular blockade in the UK.^13^ Taken together, these trends may represent attempts to balance the benefits of ‘simplicity and standardisation’ with patient-centred care and a goal of greater haemodynamic stability.^11^^,^^22^ Further research is warranted to establish optimal maintenance of anaesthesia protocols, considering not just choice of drugs and their pharmacodynamic effects, but also their pharmacokinetic properties.

While we were able to elicit greater granularity regarding maintenance regimens used by prehospital and retrieval medicine services across the UK, AU, and NZ, the majority of responding services either gave no detailed guidance on dosing for one or more of their infusion or bolus regimens, or reported no SOP or guidance for maintenance of anaesthesia post-RSI. Moreover, very few services had a protocol specific to prehospital trauma patients, suggesting a tendency to use the same maintenance regimen across heterogenous patient populations. This is consistent with the findings of Hodkinson and Poole^22^ on PHEA induction practices in the UK. Further, of the services that provided specific dosing recommendations in their anaesthesia maintenance protocols, the range of dosing between regimens of the same drug was considerable, with differences up to a factor of 10. Despite this, most services considered their maintenance protocols to be effective. While this survey did not seek to objectively assess governance and effectiveness in this area of practice, the high degree of variability in recommended regimens in contrast to the consistent perception of effectiveness warrants further consideration.

There may be several explanations for the degree of variability in content and detail of the reported maintenance of anaesthesia regimens. First, the limited literature on the topic is unclear on the goal of prehospital maintenance anaesthesia post-RSI in trauma patients—specifically whether the desired endpoint is anaesthesia or indeed sedation.^3^^,^^11^, ^12^, ^13^^,^^23^, ^24^, ^25^, ^26^, ^27^ The levels of consciousness during anaesthesia and sedation exist on a spectrum, and the definitions of each are generally accepted.^32^^,^^33^ It is common practice to aim for a lighter level of sedation and analgesia in the ICU setting, although the evidence supporting this remains limited.^34^ Extrapolating this practice of sedation to the prehospital post-RSI trauma patient in the acute phase of severe injury may be erroneous, as they are often in a hyperadrenergic state,^23^^,^^27^^,^^35^, ^36^, ^37^, ^38^ and experience further invasive procedures, ongoing pain, and exposure to stressful environments.^23^^,^^39^ Hence, avoidance of an excessive response to, and consciousness of, these noxious stimuli should be a principal clinical and ethical focus of maintenance of anaesthesia in prehospital trauma patients.^23^^,^^34^^,^^36^^,^^40^ For this reason, we have deliberately referred to this period post-RSI as anaesthesia rather than sedation. Disagreement as to the goal of hypnosis and analgesia in the post-RSI period may be one explanation for the variability in recommended dosing for maintenance regimens described in this survey. While most services in this survey considered their protocols to be effective, to our knowledge there is no literature reporting the incidence of awareness as it relates to maintenance of anaesthesia in prehospital trauma patients. Literature dealing with awareness under anaesthesia in operating theatres and the emergency department suggests an unreported rate that will only be elicited though active questioning and investigation.^40^^,^^41^ Hence, consensus on the goals of maintenance of anaesthesia (or sedation) post-RSI in prehospital trauma patients may help develop objective measures of effectiveness and governance.

Second, there are significant geographical and logistical differences between and within the services and the three countries surveyed.^5^^,^^6^^,^^42^ The time a patient remains in their care under anaesthesia may determine what drug(s) and method(s) of delivery a service or individual clinician chooses for maintenance of anaesthesia. Maintaining anaesthesia by intermittent boluses may be appropriate for a short duration, while longer periods of care may favour infusions. However, at what time point such a distinction should be drawn, and whether services are adequately considering the pharmacokinetic characteristics of the drug(s) and method(s) of delivery they are using, warrants further consideration.

Third, the induction and maintenance of anaesthesia in trauma patients provides unique clinical challenges,^21^^,^^35^^,^^43^ and these are often magnified in the prehospital setting.^9^^,^^11^, ^12^, ^13^, ^14^^,^^22^^,^^23^^,^^25^ The clinical signs conventionally cited as the means to assess adequacy of anaesthesia (e.g. heart rate, blood pressure, respiratory rate, lacrimation, pupil size, etc.) are often deranged after trauma and unreliable.^26^^,^^40^ Despite this, the majority of services in this survey recommended adjustments to their prescribed maintenance regimens based on clinical criteria. Further, the provision of adequate hypnosis and analgesia in trauma patients must be balanced against the deleterious effects of hypnotic and analgesic drugs on haemodynamic stability.^7^^,^^21^^,^^22^^,^^35^^,^^43^ Hence, the selection and dosing of hypnotic and analgesic drugs is often primarily determined by a trauma patient's haemodynamic responses.^11^^,^^21^, ^22^, ^23^^,^^26^^,^^35^ This puts haemodynamically compromised patients at higher risk of inadequate anaesthesia,^26^^,^^35^^,^^43^ with studies demonstrating significantly delayed or even no subsequent prehospital hypnosis or analgesia post-RSI, particularly in the setting of ongoing neuromuscular blockade.^23^^,^^26^^,^^27^

Fourth, there is a trend away from didactic protocol-driven care towards clinician discretion and individualised patient-centred care.^22^ While the vast majority of responding services provide physician-led PHEA,^13^^,^^42^ a smaller number of services deploy non-medical clinicians with a more limited scope of practice, and therefore may deliver more protocol-driven care with less variation. Conversely, many respondent services expressed a desire for clinicians to use their discretion to determine the appropriate drug and dosing for each individual patient, and this trend is consistent with findings regarding PHEA induction.^22^ While this approach may help address the heterogeneity of prehospital trauma patients and hence explain a degree of variation, whether dosing differences up to a factor of 10 is in keeping with pharmacokinetic and pharmacodynamic principles of safe and effective anaesthesia in this patient population requires further consideration and investigation.

Fifth, while physiological and pharmacodynamic factors are often considered, it appears less concern is given to pharmacokinetic principles. The provision of continuous, adequate anaesthesia requires maintenance of drug plasma concentrations above a minimal threshold. However, this survey demonstrated several practices that may lead to gaps in adequate plasma concentrations, or to adequate plasma concentrations not being achieved.

One such practice is dosing independent of body weight, which may lead to inadequate plasma concentrations in larger patients, as weight is an important factor determining the plasma concentration of most anaesthetic drugs for a given dose.^40^^,^^44^ A second practice is bolus-only anaesthesia maintenance. This risks intermittent troughs in plasma concentration below the threshold required for adequate anaesthesia if either the bolus dose is inadequate, the period between boluses is prolonged, or both. Further, intermittent boluses cause more rapid and varied plasma concentrations and potentially greater variations in pharmacodynamic responses. The practice of bolus-only maintenance may in part be as a result of the burden of carrying and using syringe drivers. This burden is demonstrated with several services mixing more than one drug in a single syringe to provide maintenance by continuous infusion. While this practice is controversial,^45^ it has not been shown to affect the provision of individual drugs when used in combination,^46^ and the combination of other drugs has been shown clinically to be safe and effective.^47^^,^^48^ In the prehospital setting it has the added practical and logistical benefits of reducing both clinician workload and the need to carry fewer syringe drivers.

**Fig 2:**
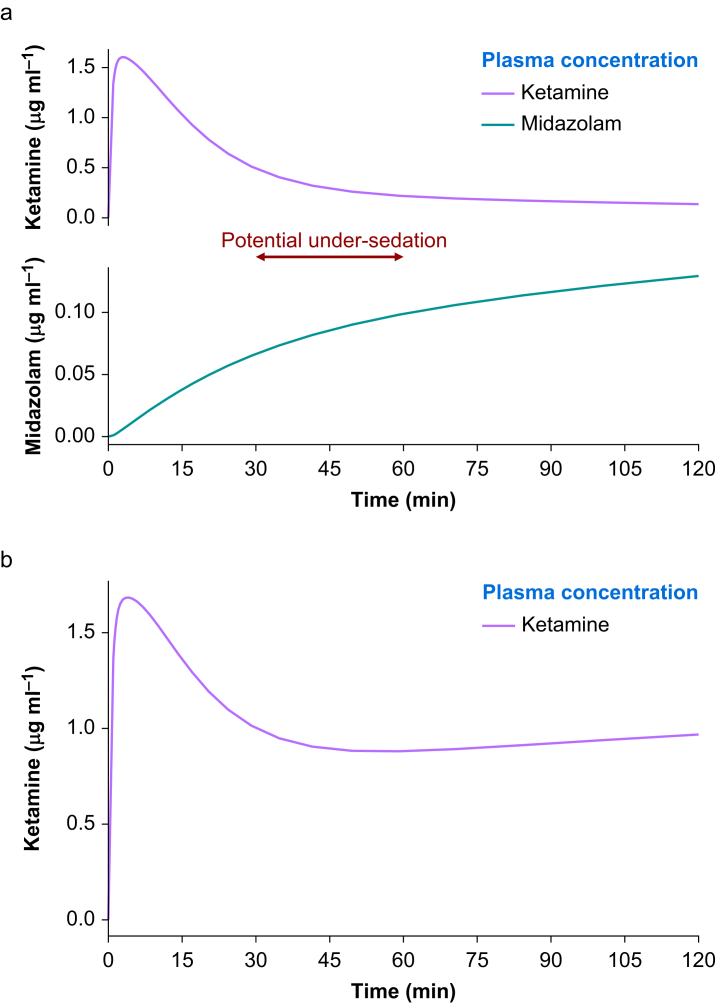
(a) Pharmacokinetic model showing plasma concentrations of ketamine and midazolam after a 2 mg kg^−1^ bolus of ketamine and a concurrent 0.1 mg kg^−1^ h^−1^ infusion of midazolam. The plasma concentration of ketamine at which a subject wakes is below 0.5 μg ml^−1^, while the plasma concentration of midazolam required for anaesthesia is above 0.1 μg ml^−1^.^50^ A period of potential under-sedation (indicated in red) occurs when the plasma concentration of ketamine drops below an adequate level before the plasma concentration of midazolam reaches an adequate level for anaesthesia. Model based on a 40-yr-old 80 kg, 180 cm male using the *stanpumpR* open-access software (https://steveshafer.shinyapps.io/stanpumpr. (b) Pharmacokinetic model showing plasma concentration of ketamine after a 2 mg kg^−1^ bolus and a concurrent 2 mg kg^−1^ h^−1^ infusion. The bolus of ketamine establishes the required plasma concentration and the infusion maintains the plasma concentration. Model based on a 40-yr-old 80 kg, 180 cm male using the *stanpumpR* open-access software (https://steveshafer.shinyapps.io/stanpumpr). (For interpretation of the references to colour in this figure legend, the reader is referred to the Web version of this article.)

A third practice is delaying the time between induction of anaesthesia and onset of maintenance, as this is recognised as a high-risk period for awareness.^40^^,^^41^^,^^43^^,^^49^ If the declining plasma concentration from the induction bolus falls below the level required for adequate anaesthesia before a subsequent bolus or continuous infusion achieves an adequate level, under-sedation and potential awareness may ensue (Fig. 2a).^40^^,^^49^ This risk appears more pronounced when longer-acting neuromuscular blocking drugs are used.^23^^,^^26^^,^^27^^,^^40^^,^^41^^,^^51^^,^^52^ A similar phenomenon may occur during the handover period of trauma patients in emergency departments. During this time, the responsibility for maintenance of anaesthesia is transferred from prehospital to hospital teams, and issues with establishing and continuing adequate anaesthesia in the emergency department is an identified problem.^39^^,^^41^^,^^51^^,^^52^ With regards to the initiation of maintenance anaesthesia in this survey, no service recommended a minimum time between induction and subsequent maintenance bolus, and only a minority of services recommended initiating a maintenance infusion immediately post-RSI. Similarly with regards to handover, no service using intermittent boluses recommended a specific timing for the last dose before handover.

A fourth practice is the use of different drugs for induction and maintenance, as this may compound the risk of awareness during this high-risk interval period. When the same drug is used for induction and maintenance by infusion, the induction dose can serve to establish an adequate plasma concentration, and the infusion can maintain it (Fig. 2b).^44^ However, when a different drug is used for maintenance, particularly when initiation is delayed, a period of potential inadequate anaesthesia can ensue (Fig. 2a). In 2009, Cowan and colleagues^13^ showed that while etomidate or ketamine was preferred for anaesthesia induction, a combination of midazolam and an opioid was preferred for maintenance. Moreover, in 2023 Hodkinson and Poole^22^ showed that while ketamine has become the exclusive hypnotic of choice for anaesthesia induction, it was only used by infusion for maintenance in 16% of services while 32% used propofol. Similarly, results from our survey showed that most services using midazolam or propofol by infusion for maintenance do not use these drugs for anaesthesia induction.

There are several limitations to this practice survey. First, it was not possible to systematically recruit all prehospital and retrieval medicine services in AU and NZ from a central registry, and therefore some eligible services may not have received an invitation to participate. Second, recruitment was limited to Anglosphere countries and hence results may not be generalisable to other countries and healthcare systems. Third, the survey questionnaire did not distinguish between adult and paediatric practice, although services may make this distinction in their protocols. Fourth, except when drugs were combined in a single syringe, the survey was not designed to elicit whether drugs were used in isolation or combination, and if in combination under what prerequisite(s) or pattern they were given. Fifth, there is a risk of social desirability bias, particularly in the responses regarding perceived effectiveness and governance. Sixth, one of the authors was also the respondent to the survey for their service, potentially adding biases to the survey and their individual responses. Seventh, there is the possibility of the Hawthorne effect, particularly given the time between conducting the survey and publication.

### Conclusions

This multinational practice survey achieved a high response rate capturing the practice of maintenance of anaesthesia post-RSI across a range of prehospital and retrieval medicine services operating within similar healthcare systems. The survey results broadly support the hypothesis that among services that provide maintenance of anaesthesia to prehospital trauma patients post-RSI, there was considerable variability in both the content and detail of protocols governing this practice. Despite considerable variation in practice between services, most services perceived their own practice to be effective. While these results do not allow for specific conclusions and recommendations to help guide clinicians and services on appropriate maintenance protocols, they do highlight the need for further consideration of practice and research in this area. Areas for further consideration include consensus on the specific aims of post-RSI maintenance in prehospital trauma patients, and a greater emphasis on the pharmacokinetics of the drugs used to achieve these aims. Consensus around goals for post-RSI maintenance would assist with establishing objective measures of effectiveness and with governance in this area of practice. More research is needed to establish the optimal choice of drugs, appropriate dosing, and means of delivery, and the clinical factors that may best determine requisite adjustments to these variables in prehospital trauma patients. It is hoped that this practice survey will act as a catalyst for further discussion and research in this important aspect of patient care.

## Authors’ contributions

Study conception and design: BS, ZP

Survey design, data acquisition, and analysis: BS

Data interpretation: BS, ZP

Drafting of manuscript and revisions: BS, ZP

Both authors approved the final version of the paper.

## Declarations of interest

The authors declare that they have no conflicts of interest.
