# Supplementary material for: Maintenance of prehospital anaesthesia in trauma patients: inconsistencies and variability in practice
Source: BJA Open. 2025 Jan 9;13:100366. doi: 10.1016/j.bjao.2024.100366 (PMC11764628; doi:10.1016/j.bjao.2024.100366)
Supplement: Multimedia component 1 [file mmc1.pdf]

APPENDICIES

Appendix 1. Survey questions

**Context**	
Name of Service	______________________
Country of Practice	______________________

**Standard Operating Procedures**	
Does your service provide specific guidance to clinicians regarding anaesthesia/sedation post-PHEA in prehospital trauma patients, in the form of a guideline/protocol/SOP?<br><br>(this may be contained within a broader guideline/protocol/SOP addressing PHEA, and may encompass prehospital medical and/or intubated interhospital patients)	◯ Yes<br>◯ No
Is this guidance specific to prehospital trauma patients, or does it also cover prehospital medical patients and/or intubated interhospital transfer patients?	◯ Specific to prehospital trauma patients<br>◯ Also for prehospital medical and/or intubated interhospital patients (as well as prehospital trauma patients)
Is this guidance	◯ Separate to your PHEA guideline/protocol/SOP<br>◯ Contained within your PHEA guideline/protocol/SOP
Does this guideline/protocol/SOP involve	◯ Intermittent bolus sedation/anaesthesia/paralysis?<br>◯ Continuous infusion sedation/anaesthesia/paralysis?<br>◯ A combination (eg continuous infusion with intermittent boluses as needed, a choice of either etc)

Intermittent Bolus

If intermittent bolus, does this guideline/protocol/SOP nominate or specify a choice of drug(s)?	◯ Yes<br>◯ No
Which drug(s) are specified for intermittent bolus?	☐ Ketamine<br>☐ Midazolam<br>☐ Propofol<br>☐ Fentanyl<br>☐ Morphine<br>☐ Rocuronium<br>☐ Other

Intermittent Bolus: Ketamine

Is Ketamine also used for induction?	◯ Yes<br>◯ No
Is there a recommended intermittent bolus dose of Ketamine for ongoing anaesthesia/sedation?	◯ Yes<br>◯ No
If Yes, what is the dose (or range)?	__________________________
Is there a recommended dosing interval for intermittent boluses of Ketamine?	◯ Yes<br>◯ No
What is the recommended dosing interval for Ketamine ? (in mins)	__________________________
Is there specific guidance for the timing of the first dose of Ketamine post-RSI?	◯ Yes<br>◯ No
If Yes, when is this first dose of Ketamine recommended?	__________________________
Is there specific guidance for the timing of the last dose of Ketamine prior to handover?	◯ Yes<br>◯ No
If Yes, when is this last dose of Ketamine recommended?	__________________________

Intermittent Bolus: Midazolam

Is Midazolam also used for induction?	◯ Yes<br>◯ No
Is there a recommended intermittent bolus dose of Midazolam for ongoing anaesthesia/sedation?	◯ Yes<br>◯ No
If Yes, what is the dose (or range)?	________________________
Is there a recommended dosing interval for intermittent boluses of Midazolam?	◯ Yes<br>◯ No
What is the intermittent dosing interval for Midazolam? (in mins)	________________________
Is there specific guidance for the timing of the first dose of Midazolam post-RSI?	◯ Yes<br>◯ No
If Yes, when is this first dose of Midazloam recommended?	________________________
Is there specific guidance for the timing of the last dose of Midazolam prior to handover?	◯ Yes<br>◯ No
If Yes, when is this last dose of Midazolam recommended?	________________________

Intermittent Bolus: Propofol

Is Propofol also used for induction?	◯ Yes<br>◯ No
Is there a recommended intermittent bolus dose of Propofol for ongoing anaesthesia/sedation?	◯ Yes<br>◯ No
If Yes, what is the dose (or range)?	________________________
Is there a recommended dosing interval for intermittent boluses of Propofol?	◯ Yes<br>◯ No
What is the intermittent dosing interval for Propofol? (in mins)	________________________
Is there specific guidance for the timing of the first dose of Propofol post-RSI?	◯ Yes<br>◯ No
If Yes, when is this first dose of Propofol recommended?	________________________
Is there specific guidance for the timing of the last dose of Propofol prior to handover?	◯ Yes<br>◯ No
If Yes, when is this last dose of Propofol recommended?	________________________

Intermittent Bolus: Fentanyl

Is Fentanyl also used for induction?	◯ Yes<br>◯ No
Is there a recommended intermittent bolus dose of Fentanyl for ongoing anaesthesia/sedation	◯ Yes<br>◯ No
If Yes, what is the dose (or range)?	________________________
Is there a recommended dosing interval for intermittent boluses of Fentanyl?	◯ Yes<br>◯ No
What is the intermittent dosing interval for Fentanyl? (in mins)	________________________
Is there specific guidance for the timing of the first dose of Fentanyl post-RSI?	◯ Yes<br>◯ No
If Yes, when is this first dose of Fentanyl recommended?	________________________
Is there specific guidance for the timing of the last dose of Fentanyl prior to handover?	◯ Yes<br>◯ No
If Yes, when is this last dose of Fentanyl recommended?	________________________

Intermittent Bolus: Morphine

Is Morphine also used for induction?	◯ Yes<br>◯ No
Is there a recommended intermittent bolus dose of Morphine for ongoing anaesthesia/sedation?	◯ Yes<br>◯ No
If Yes, what is the dose (or range)?	________________________
Is there a recommended dosing interval for intermittent boluses of Morphine?	◯ Yes<br>◯ No
What is the intermittent dosing interval for Morphine? (in mins)	________________________
Is there specific guidance for the timing of the first dose of Morphine post-RSI?	◯ Yes<br>◯ No
If Yes, when is this first dose of Morphine recommended?	________________________
Is there specific guidance for the timing of the last dose of Morphine prior to handover?	◯ Yes<br>◯ No
If Yes, when is this last dose of Morphine recommended?	________________________

**Intermittent Bolus: Rocuronium**	
Is Rocuronium also used for induction?	◯ Yes<br>◯ No
Is there a recommended intermittent bolus dose of Rocuronium for ongoing anaesthesia/sedation?	◯ Yes<br>◯ No
If Yes, what is the dose (or range)	__________________________
Is there a recommended dosing interval for intermittent boluses of Rocuronium?	◯ Yes<br>◯ No
What is the intermittent dosing interval for Rocuronium? (in mins)	__________________________
Is there specific guidance for the timing of the first dose of Rocuronium post-RSI?	◯ Yes<br>◯ No
If Yes, when is this first dose of Rocuronium recommended?	__________________________
Is there specific guidance for the timing of the last dose of Rocuronium prior to handover?	◯ Yes<br>◯ No
If Yes, when is this last dose of Rocuronium recommended?	__________________________

**Intermittent Bolus: Other Drug 1**	
Other 1, please specify drug name	__________________________
Is [other_intermittent_drug_1] also used for induction?	◯ Yes<br>◯ No
Is there a recommended intermittent bolus dose of [other_intermittent_drug_1] for ongoing anaesthesia/sedation?	◯ Yes<br>◯ No
If yes, what is the dose (or range) of [other_intermittent_drug_1]?	__________________________
Is there a recommended dosing interval for intermittent boluses of [other_intermittent_drug_1]?	◯ Yes<br>◯ No
What is the recommended dosing interval for [other_intermittent_drug_1]? (in mins)	__________________________
Is there specific guidance for the timing of the first dose of [other_intermittent_drug_1] post-RSI?	◯ Yes<br>◯ No
If yes, when is this first dose of [other_intermittent_drug_1] recommended?	__________________________
Is there specific guidance for the timing of the last dose of [other_intermittent_drug_1] prior to handover?	◯ Yes<br>◯ No
If yes, when is this last dose of [other_intermittent_drug_1] recommended?	__________________________

Intermittent Bolus: Other Drug 2

Other 2, please specify drug name	______________________
Is [other_intermittent_drug_2] also used for induction?	◯ Yes<br>◯ No
Is there a recommended intermittent bolus dose of [other_intermittent_drug_2] for ongoing anaesthesia/sedation?	◯ Yes<br>◯ No
If yes, what is the dose (or range) of [other_intermittent_drug_2]?	______________________
Is there a recommended dosing interval for intermittent boluses of [other_intermittent_drug_2]?	◯ Yes<br>◯ No
What is the recommended dosing interval for [other_intermittent_drug_2]? (in mins)	______________________
Is there specific guidance for the timing of the first dose of [other_intermittent_drug_2] post-RSI?	◯ Yes<br>◯ No
If yes, when is this first dose of [other_intermittent_drug_2] recommended?	______________________
Is there specific guidance for the timing of the last dose of [other_intermittent_drug_2] prior to handover?	◯ Yes<br>◯ No
If yes, when is this last dose of [other_intermittent_drug_2] recommended?	______________________

Intermittent Bolus: Other Drug 3

Other 3, please specify drug name	______________________
Is [other_intermittent_drug_3] also used for induction?	◯ Yes<br>◯ No
Is there a recommended intermittent bolus dose of [other_intermittent_drug_3] for ongoing anaesthesia/sedation?	◯ Yes<br>◯ No
If yes, what is the dose (or range) of [other_intermittent_drug_3]?	______________________
Is there a recommended dosing interval for intermittent boluses of [other_intermittent_drug_3]?	◯ Yes<br>◯ No
What is the recommended dosing interval for [other_intermittent_drug_3]? (in mins)	______________________
Is there specific guidance for the timing of the first dose of [other_intermittent_drug_3] post-RSI?	◯ Yes<br>◯ No
If yes, when is this first dose of [other_intermittent_drug_3] recommended?	______________________

Is there specific guidance for the timing of the last dose of [other_intermittent_drug_3] prior to handover?	◯ Yes<br>◯ No
If yes, when is this last dose of [other_intermittent_drug_3] recommended?	______________________________

Intermittent Adjustments

Are recommendations given to adjust the drug choice/dose/timing of intermittent boluses based on clinical parameters?	◯ Yes<br>◯ No
If yes, what adjustments, and based on what parameters?<br><br>e.g. reduced dose in hypovolaemia	____________________________________

Continuous Infusion

If continuous infusion, does this guideline/protocol/SOP nominate or specify a choice of drug(s)?	◯ Yes<br>◯ No
Which drug(s) are used for continuous infusion?	☐ Ketamine<br>☐ Midazolam<br>☐ Propofol<br>☐ Fentanyl<br>☐ Morphine<br>☐ Rocuronium<br>☐ Other

Continuous Infusion: Ketamine

Is Ketamine also used for induction?	◯ Yes<br>◯ No
What is the rate (or range) for the continuous infusion of Ketamine?	__________________________
When is the infusion of Ketamine started?	◯ immediately post-induction<br>◯ between induction and loading<br>◯ on/after loading into transport vehicle<br>◯ at clinician's discretion<br>◯ Other
If Other, please specify	________________________________

Continuous Infusion: Midazolam

Is Midazolam also used for induction?	◯ Yes<br>◯ No
What is the rate (or range) for the continuous infusion of Midazolam?	__________________________
When is the infusion of Midazolam started?	◯ immediately post-induction<br>◯ between induction and loading<br>◯ on/after loading into transport vehicle<br>◯ at clinician's discretion<br>◯ Other
If Other, please specify	________________________________

Continuous Infusion: Propofol

Is Propfol also used for induction?

◯ Yes
◯ No

What is the rate (or range) for the continuous infusion of Propofol?

____

When is the infusion of Propofol started?

◯ immediately post-induction
◯ between induction and loading
◯ on/after loading into transport vehicle
◯ at clinician's discretion
◯ Other

If Other, please specify

____

Continuous Infusion: Fentanyl

Is Fentanyl also used for induction?

◯ Yes
◯ No

What is the rate (or range) for the continuous infusion of Fentanyl?

____

When is the infusion of Fentanyl started?

◯ immediately post-induction
◯ between induction and loading
◯ on/after loading into transport vehicle
◯ at clinician's discretion
◯ Other

If Other, please specify

____

Continuous Infusion: Morphine

Is Morphine also used for induction?

◯ Yes
◯ No

What is the rate (or range) for the continuous infusion of Morphine?

____

When is the infusion of Morphine started?

◯ immediately post-induction
◯ between induction and loading
◯ on/after loading into transport vehicle
◯ at clinician's discretion
◯ Other

If Other, please specify

____

Continuous Infusion: Rocuronium

Is Rocuronium also used for induction?	◯ Yes<br>◯ No
What is the rate (or range) for the continuous infusion of Rocuronium?	______________________________
When is the infusion of Rocuronium started?	◯ immediately post-induction<br>◯ between induction and loading<br>◯ on/after loading into transport vehicle<br>◯ at clinician's discretion<br>◯ Other
If Other, please specify	_____________________________________

Continuous Infusion: Other Drug 1

Please name this Other drug 1 used by continuous infusion	______________________________
Is [continuous_other_name_1] also used for induction?	◯ Yes<br>◯ No
What is the rate (or range) for the continuous infusion of [continuous_other_name_1]?	______________________________
When is the infusion of [continuous_other_name_1] started?	◯ immediately post-induction<br>◯ between induction and loading<br>◯ on/after loading into transport vehicle<br>◯ at clinician's discretion<br>◯ Other
If at another time, please specify when	_____________________________________

Continuous Infusion: Other Drug 2

Please name this Other drug 2 used by continuous infusion	______________________________
Is [continuous_other_name_2] also used for induction?	◯ Yes<br>◯ No
What is the rate (or range) for the continuous infusion of [continuous_other_name_2]?	______________________________
When is the infusion of [continuous_other_name_2] started?	◯ immediately post-induction<br>◯ between induction and loading<br>◯ on/after loading into transport vehicle<br>◯ at clinician's discretion<br>◯ Other
If at another time, please specify when	_____________________________________

Continuous Infusion: Other Drug 3

Please name this Other drug 3 used by continuous infusion

____

Is [continuous_other_name_3] also used for induction?

◯ Yes
◯ No

What is the rate (or range) for the continuous infusion [continuous_other_name_3]?

____

When is the infusion of [continuous_other_name_3] started?

◯ immediately post-induction
◯ between induction and loading
◯ on/after loading into transport vehicle
◯ at clinician's discretion
◯ Other

If at another time, please specify when

____

Continuous Infusion: Adjustments

Are recommendations given to adjust the drug choice/infusion rate of the continuous infusion(s) based on clinical parameters?

◯ Yes
◯ No

If Yes, what adjustments, and based on what parameters?

e.g. reduced rate in hypovolaemia

____

Effectiveness of Practice

Governance

In general, do you believe your service's practice is effective in providing uninterrupted, adequate levels of anaesthesia/sedation for prehospital trauma patients post-PHEA?	◯ Ineffective<br>◯ Somewhat ineffective<br>◯ Neutral<br>◯ Effective<br>◯ Very Effective
In general, how much time/discussion in governance meetings is dedicated to post-RSI sedation practices relative to the RSI itself?	◯ No time/discussion<br>◯ Some time/discussion<br>◯ About the same amount of time/discussion<br>◯ More time/discussion<br>◯ Considerably more time/discussion

Additional Comments

Additional Comments	____
